# Supplementary material for: Conceptualizing the process of experiential acceptance during an episode of anxiety: A qualitative study of individuals with prior experience in mindfulness- and acceptance-based interventions
Source: PLoS One. 2026 Nov 10;21(11):e0344230. doi: 10.1371/journal.pone.0344230 (PMC13750539; doi:10.1371/journal.pone.0344230)
Supplement: Supplementary file 1 [file pone.0344230.s001.docx]

S1 Appendix. Interview Guide and Description of its Evolution

# Evolution and Iterative Refinement of the Semi-Structured Interview Guide

The interview guide retained a stable core focused on participants’ understanding and use of experiential acceptance, the detailed description of concrete anxiety-provoking episodes, the operations involved in acceptance, and its facilitators, obstacles, conditions, and effects. During the initial interviews (P1–P5), broader questions concerning the general manifestations of anxiety and participants’ inner dialogue were also explored. These questions were progressively reduced or discontinued because they contributed limited information to the conceptualization of experiential acceptance.

From P6 onward, follow-up probes became more specifically focused on how participants concretely engaged with discomfort and on refining the properties, conditions, and limits of the emerging categories. From P16 onward, interviews increasingly sought boundary cases, counterexamples, ambiguities and misuses of acceptance, variations according to experience, and conditions requiring a flexible rather than continuous contact with internal experience, thereby further refining the emerging conceptual model.

**Table 1. Evolution of the Semi-Structured Interview Guide Across Data Collection Phases**

| Data collection phase | Participants | Main interview guide content | Specific developments and follow-up probes | Analytic function |
| --- | --- | --- | --- | --- |
| 1- Initial exploration | P1–P5 | Participants’ experience of anxiety, usual regulation strategies, understanding of acceptance, contexts of use, description of a concrete acceptance episode, unfolding of the process, barriers, facilitators, and perceived effects | Broad exploration of anxiety. These questions were progressively reduced because they yielded limited information relevant to the conceptual development of experiential acceptance. | Identification of the emerging categories and their initial properties. |
| 2- Further development of the categories | From P6 onward | Core guide refocused on concrete descriptions of acceptance episodes | More targeted probes on how participants engaged with discomfort, attentional, bodily, and behavioral operations, non-reactivity, maintenance of contact with experience, facilitators, barriers, conditions, and limits | Refinement of the properties, relationships, and conditions of the emerging categories |
| 3- Refining the emerging model | From P16 onward | Description of concrete episodes and targeted exploration of the emerging categories | Targeted exploration of boundary cases, counterexamples, pseudo-acceptance, variations according to experience, the influence of anxiety intensity, flexible contact with internal experience, and the distinction between contextually appropriate action and anxiety-driven action. | Refinement of category boundaries, exploration of negative cases and variations, and strengthening the explanatory scope of the emerging model. |

# Original French Interview Guide

#### Tout d’abord merci d’avoir accepté de participer à cette étude qui porte sur le vécu de l’acceptation des émotions. Il n’y a pas de bonnes ou mauvaises réponses, pas de réponse spécifique attendue, ce qui m’intéresse c’est d’explorer votre expérience et comment cela se passe pour vous. Vous serez amené.e à parler de vous, de vos émotions notamment de votre anxiété. Vous pouvez interrompre l’entretien dès que vous en ressentez le besoin.

#### Pour des fins d’analyses, j’ai besoin d’enregistrer notre entretien. Tout ce que vous me direz ici sera anonymisé et votre nom n’apparaîtra pas dans mes travaux.

#### Êtes-vous d’accord pour continuer ?

## Introduction – vous et les interventions de pleine conscience

### - Quel(s) type(s) d’intervention(s) basée sur la pleine conscience avez-vous reçu ?

### - Quand l’avez-vous reçue ?

### - (Si plusieurs) Quelle est la date (année) de la 1ère intervention que vous avez suivie ? et la dernière ?

## Thème 1. Expérience d’une émotion : l’anxiété

#### Cette étude porte plus spécifiquement sur l’anxiété et nous nous intéressons à la manière dont vous acceptez cette émotion, plutôt que de la combattre ou l’éviter. Pour m’assurer que nous partageons la même compréhension de l’anxiété, je vais vous en proposer une définition et vous me direz ensuite si elle correspond à votre propre conception : « l’anxiété est une émotion naturelle, souvent persistante, liée à des préoccupations futures ou à des incertitudes. Elle peut se manifester par des pensées récurrentes d’inquiétude, de peur ou des préoccupations. Elle peut être accompagnée de symptômes physiques similaires à ceux du stress (palpitations, tensions musculaires, difficultés à respirer, etc.) »

#### Est-ce que cela correspond à votre conception de l’anxiété ?

1.	Quel est votre rapport à l’anxiété ?

#### Note de l’intervieweur: Entretiens initiaux uniquement (questions ensuite abandonnées car elles apportaient peu d’éléments utiles à la conceptualisation de l’acceptation expérientielle)

### Qu’est-ce qui vous stresse/rend anxieux.se/préoccupe ?

### Comment cela se manifeste ?

### Aspects cognitifs (e.g., inquiétudes, pensées négatives),

### Aspects physiologiques (e.g., neurovégétatif, sensations corporelles),

### Aspects comportementaux (e.g., tendances à l’action)

### Est-ce supportable pour vous de ressentir de l’anxiété ? Avez-vous l’impression que c’est particulièrement difficile pour vous lorsque vous vous sentez anxieux.se ?

## Thème 2. Stratégies habituelles de régulation émotionnelle et acceptation

2.	Habituellement, quand vous vous sentez anxieux.se, que faites-vous ? OU Comment réagissez-vous généralement à votre anxiété lorsqu’elle survient ?

3.	Vous avez récemment suivi une (nom de l’intervention), basée sur la pleine conscience et l’acceptation des émotions. Qu’avez-vous appris pour faire face à votre anxiété ?

4.	Qu’est-ce qu’accepter/s’ouvrir/faire de la place à ses expériences internes / émotions signifie pour vous ?

5.	Et comment vous faites concrètement pour accepter ?

#### Note de l’intervieweur: Suivi de la question 8 pour obtenir une illustration concrète.

## Thème 3. Motivation et obstacles à l’utilisation d’une stratégie

6.	Dans quel(s) cas/contexte et pour quelle(s) émotion(s) utilisez-vous l’acceptation ?

7.	Pourquoi ne l’utilisez-vous pas à un autre moment ? OU Qu’est-ce qui fait que vous ne choisissez pas d’accepter votre anxiété dans certains contextes ?

## Thème 4. Expérience du vécu d’acceptation de l’anxiété

8.	Pouvez-vous me décrire un moment où vous êtes parvenu à accepter votre anxiété (plutôt que de la combattre ou l’éviter) ? Comment cela s’est-il passé en vous ?

9.	Que ressentez-vous pendant que vous acceptez (votre anxiété) ? OU Comment savez-vous que vous y êtes ? (Que vous êtes en train d’accepter)

10.	Qu’est-ce qui fait que ça ne marche pas ? Quels obstacles pouvez-vous rencontrer ? Y a-t-il des situations où il est plus difficile d’accepter ?

11.	Et a contrario qu’est-ce qui aide dans cette démarche ?

12.	A quel point est-ce facile ou difficile pour vous à mettre en œuvre ?

13.	Avez-vous un dialogue intérieur qui vous guide pendant votre expérience d’acceptation ?

#### Note de l’intervieweur: Entretiens initiaux uniquement (question ensuite abandonnée en raison de la difficulté des participants à fournir des réponses suffisamment précises et exploitables pour l’analyse).

14.	Comment votre pratique a-t-elle évolué avec le temps, l’expérience ?

## Thème 5. Effets de l’acceptation

15.	Comment savez-vous que ça a marché ? Que vous avez pu accepter l’anxiété ?

16.	Comment vous sentez-vous après avoir accepté votre émotion d’anxiété ?

17.	Comment la pratique de l’acceptation de votre anxiété a-t-elle influencé votre vie émotionnelle et votre bien-être ?

18.	Qu’avez-vous appris sur vous-même depuis que vous pratiquez l’acceptation ?

## Thème 6. Aller plus loin

19.	Si quelqu’un vous disait qu’il ne parvient pas à accepter son émotion d’anxiété, quels conseils lui donneriez-vous pour le faire ?

## Clôture de l’entretien

#### Y a-t-il autre chose que vous aimeriez partager concernant votre expérience de l’acceptation ?

#### Cet entretien de recherche est maintenant terminé. Je vous remercie pour vos réponses et le temps que vous y avez consacré.

# English Translation of the Original French Interview Guide

#### Interviewer note: The interviews were conducted in French using the original French-language interview guide. The version presented below is an English translation provided for publication.

#### First, thank you for agreeing to participate in this study, which explores the lived experience of emotional acceptance. There are no right or wrong answers, and no specific response is expected. What interests me is your own experience and how acceptance unfolds for you. During this interview, you will be invited to talk about yourself, your emotions, and particularly your experiences of anxiety. You may stop the interview at any time if you wish.

#### For the purpose of data analysis, I need to audio-record our interview. Everything you say will be anonymized, and your name will not appear in any publications arising from this research.

Do you agree to continue?

## Introduction – You and Mindfulness-based Interventions

### What type(s) of mindfulness-based intervention(s) have you participated in?

### When did you receive this intervention?

### If you participated in more than one intervention:

### When did you first participate in a mindfulness-based intervention?

### What was the most recent one?

## Theme 1. Experience of Anxiety

This study focuses specifically on anxiety and on how people accept this emotion rather than fight or avoid it. To ensure that we share a common understanding of anxiety, I will first provide a definition, after which I will ask whether it corresponds to your own understanding.

Anxiety is a natural emotion that is often persistent and related to future concerns or uncertainty. It may involve recurrent thoughts of worry, fear, or apprehension and may be accompanied by physical symptoms similar to those associated with stress (e.g., palpitations, muscle tension, shortness of breath).

Does this definition correspond to your understanding of anxiety?

1. What is your relationship with anxiety?

#### Interviewer note: Initial interviews only (later discontinued because they yielded limited information for the conceptualization of experiential acceptance)

- What tends to make you anxious, stressed, or worried?

- How does anxiety manifest itself for you?

* Cognitive aspects (e.g., worries, negative thoughts)

* Physiological aspects (e.g., autonomic arousal, bodily sensations)

* Behavioral aspects (e.g., action tendencies)

- How tolerable is it for you to experience anxiety? Do you find anxiety particularly difficult to experience?

## Theme 2. Usual Emotion Regulation Strategies and Acceptance

2. When you feel anxious, what do you usually do? OR
3. How do you generally respond when anxiety arises?
4. You recently participated in a [name of intervention] based on mindfulness and emotional acceptance. What did you learn about dealing with anxiety?
5. What does accepting, opening up to, or making space for your internal experiences or emotions mean to you?
6. And how do you actually do that in practice?

#### Interviewer note: Followed by Question 8 to obtain a concrete illustration.

## Theme 3. Motivation and Barriers to Using Acceptance

7. In what situations, contexts, or for which emotions do you use acceptance?
8. Why do you not use acceptance in other situations? OR
   What prevents you from choosing acceptance for your anxiety in certain situations?

## Theme 4. Lived Experience of Accepting Anxiety

9. Can you describe a specific occasion when you were able to accept your anxiety rather than fight or avoid it? How did this unfold within your experience?
10. What do you experience while you are accepting your anxiety? OR
11. How do you know that you are actually accepting it?
12. What makes it not work? What obstacles do you encounter? Are there situations in which accepting your anxiety is more difficult?
13. Conversely, what helps you engage in acceptance?
14. How easy or difficult is it for you to put acceptance into practice?
15. Do you have an inner dialogue that guides you during the acceptance process?

#### Interviewer note: Initial interviews only (the question was subsequently discontinued because participants had difficulty providing sufficiently precise responses for meaningful analysis).

16. How has your practice of acceptance evolved over time and with experience?

## Theme 5. Effects of Acceptance

17. How do you know that acceptance has worked? How do you know that you have accepted your anxiety?
18. How do you feel after accepting your anxiety?
19. How has practicing acceptance of anxiety influenced your emotional life and your overall well-being?
20. What have you learned about yourself since you began practicing acceptance?

## Theme 6. Looking Beyond your Own Experience

21. If someone told you they were unable to accept their anxiety, what advice would you give them?

## Closing the Interview

#### Is there anything else you would like to share about your experience of acceptance?

#### This concludes the interview. Thank you very much for your participation, your time, and your thoughtful responses.
